# 

**Published:** 1895-11-02

**Authors:** 


					A J.A1U JJUU1 liilij,
O
AiiSOLU 1'tLV FUKH, therefore BHST.? " iTOTiiniinraiajprero;
CADBURYS ^ ww ir^ CADBURY'S
COCOA ^wT M COCOA
' Of absolute purity and freedom from alkali." )??> <-?^ <?Hi nH " The Typical Cocoa of Enelish Manufacture-
?Braithwaite. Absolutely Pure."?The Analyst. ^ ^
D\T
ijjiivCiPBiii'.ttiur
RWICH HOSPl TAL
No. 475. ? Vol. xix. WITH EXTRA NURSING SUPPLEMENT.
Saturday,
Nov. 2nd, 1895.
[Registered at the General Post Office as a Newspaper for Monthly Parts, 6d.; "post free, 8d.
transmission in the United Kingdom and Abroad,]
, JIUO
. post .
Ditto per Annum, 8s.6d., post free.

				

